# Supplementary material for: Integrated Analysis of Germline and Tumor DNA Identifies New Candidate Genes Involved in Familial Colorectal Cancer
Source: Cancers (Basel). 2019 Mar 13;11(3):362. doi: 10.3390/cancers11030362 (PMC6468873; doi:10.3390/cancers11030362)
Supplement: Supplementary file 1 [file cancers-11-00362-s001.pdf]

Supplementary Materials: Integrated Analysis of Germline and Tumor DNA Identifies New Candidate Genes Involved in Familial Colorectal Cancer

Marcos Díaz-Gay, Sebastià Franch-Expósito, Coral Arnau-Collell, Solip Park, Fran Supek, Jenifer Muñoz, Laia Bonjoch, Anna Gratacós-Mulleras, Paula A. Sánchez-Rojas, Clara Esteban-Jurado, Teresa Ocaña, Miriam Cuatrecasas, Maria Vila-Casadesús, Juan José Lozano, Genis Parra, Steve Laurie, Sergi Beltran, EPICOLON Consortium, Antoni Castells, Luis Bujanda, Joaquín Cubiella, Francesc Balaguer and Sergi Castellví-Bel

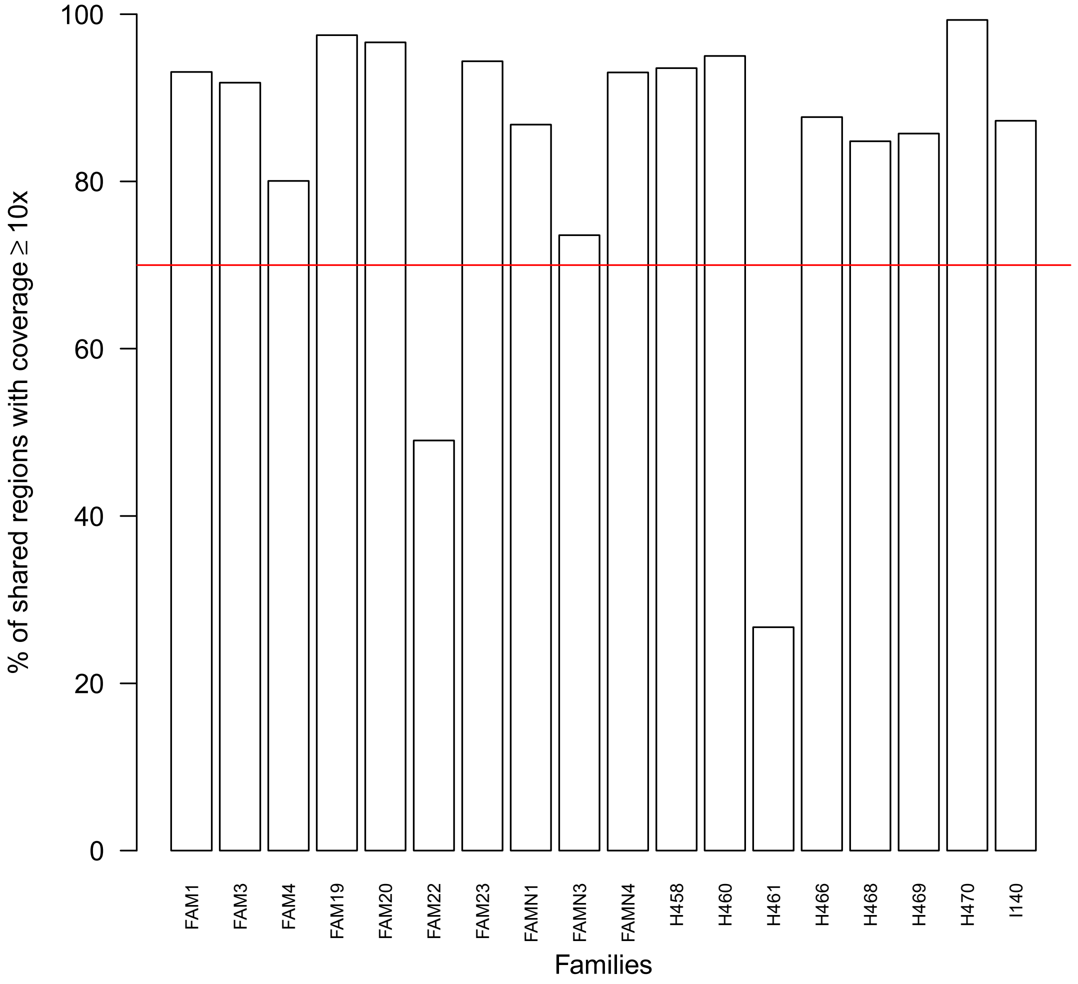

Figure S1. Histogram representing the percentage of genomic regions with a high-quality value of coverage (≥10×) with respect to all shared sequenced regions for each of the germline-tumor paired samples. Horizontal red line indicates sample filtering threshold (≥70% of shared regions with coverage above 10×).

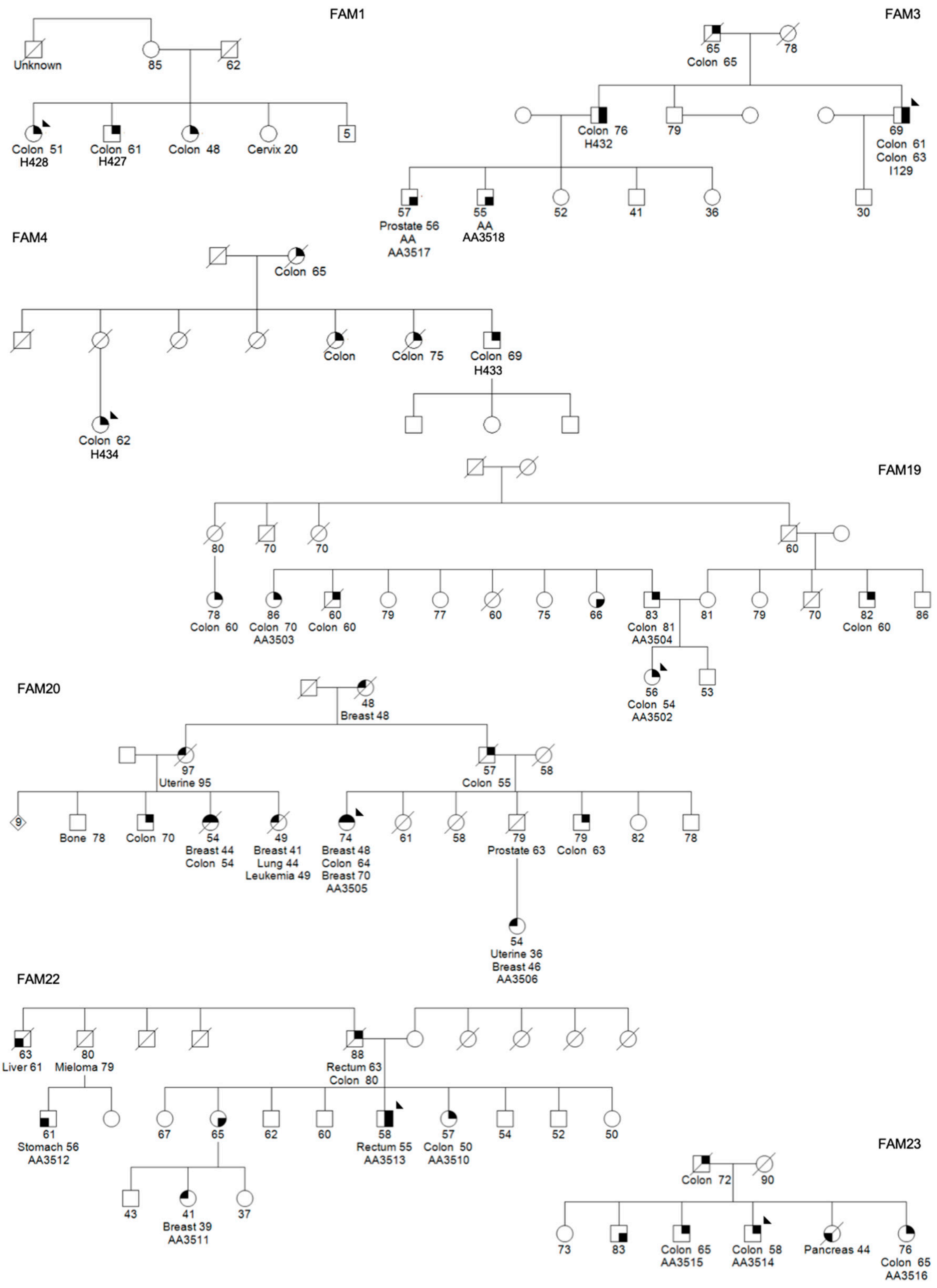
FAM1
Unknown
85
62
Colon 51
H428
Colon 61
H427
Colon 48
Cervix 20
5
FAM3
65
Colon 65
78
Colon 76
H432
79
69
Colon 61
Colon 63
I129
57
Prostate 56
AA
AA3517
55
AA
AA3518
52
41
36
30
FAM4
Colon 65
Colon
Colon 75
Colon 69
H433
Colon 62
H434
FAM19
80
70
70
60
78
Colon 60
86
Colon 70
AA3503
60
Colon 60
79
77
60
75
66
83
Colon 81
AA3504
81
79
70
82
Colon 60
86
56
Colon 54
AA3502
53
FAM20
48
Breast 48
97
Uterine 95
57
Colon 55
58
9
Bone 78
Colon 70
54
Breast 44
Colon 54
49
Breast 41
Lung 44
Leukemia 49
74
Breast 48
Colon 64
Breast 70
AA3505
61
58
79
Prostate 63
79
Colon 63
82
78
54
Uterine 36
Breast 46
AA3506
FAM22
63
Liver 61
80
Mieloma 79
88
Rectum 63
Colon 80
61
Stomach 56
AA3512
67
65
62
60
58
Rectum 55
AA3513
57
Colon 50
AA3510
54
52
50
43
41
Breast 39
AA3511
37
FAM23
Colon 72
90
73
83
Colon 65
AA3515
Colon 58
AA3514
Pancreas 44
76
Colon 65
AA3516

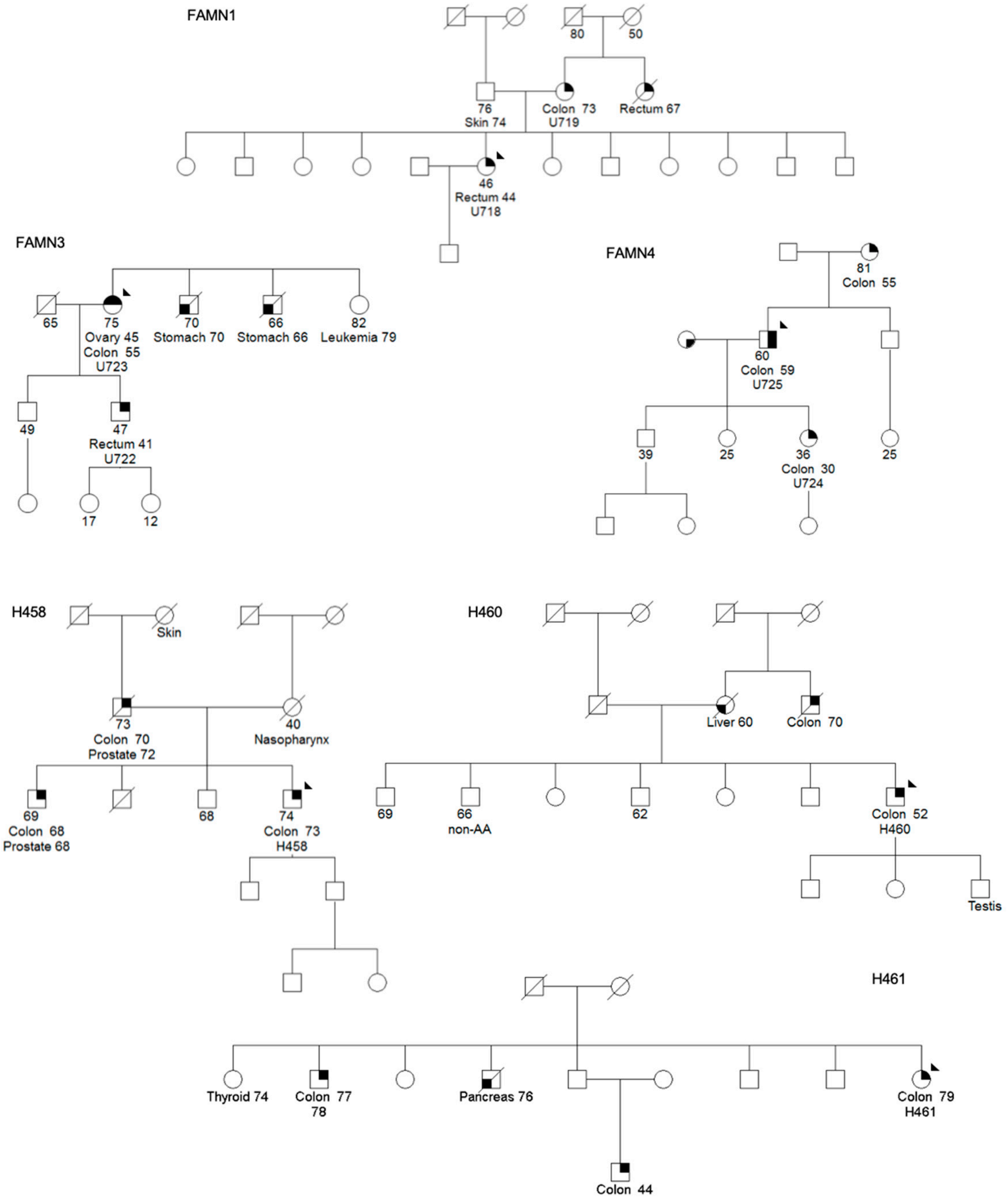
FAMN1
80
50
76
Skin 74
Colon 73
U719
Rectum 67
46
Rectum 44
U718
FAMN3
65
75
Ovary 45
Colon 55
U723
70
Stomach 70
66
Stomach 66
82
Leukemia 79
49
47
Rectum 41
U722
17
12
FAMN4
81
Colon 55
60
Colon 59
U725
39
25
36
Colon 30
U724
25
H458
Skin
73
Colon 70
Prostate 72
40
Nasopharynx
69
Colon 68
Prostate 68
68
74
Colon 73
H458
H460
Liver 60
Colon 70
69
66
non-AA
62
Colon 52
H460
Testis
H461
Thyroid 74
Colon 77
78
Pancreas 76
Colon 79
H461
Colon 44

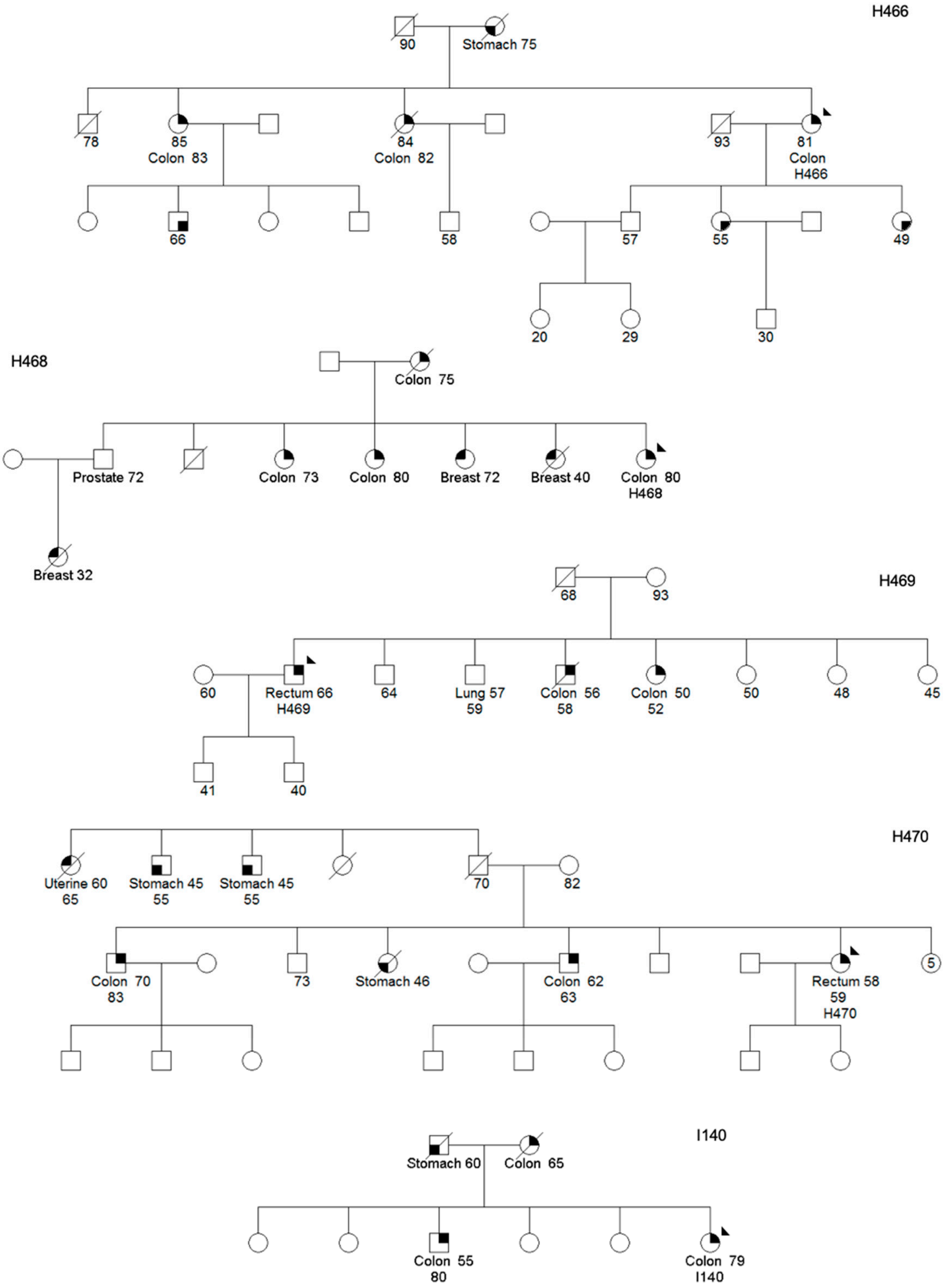

Figure S2. Pedigrees of the 18 families included in the study. Sample selected for germline and tumor whole-exome sequencing is indicated with an arrow. Filled symbols indicate affected for colorectal cancer (upper right quarter), adenoma/s (lower right quarter), gynecological cancer (ovary, uterine or breast cancer) (upper left quarter) and liver, stomach or pancreatic cancer (lower left quarter). Other cancer types are indicated in text with no symbol. IDs from samples undergoing germline whole-exome sequencing are also shown. AA/on-AA, advanced adenoma/non-advanced adenoma.

Table S1. Description of germline copy number variants detected after calling with CoNIFER and ExomeDepth.

Sample	CNV Class	Calling Tool	Location	Length (bp)	DGV Freq.	EPICOLON Freq.	Genes Included	Encoded Proteins Function/OMIM
AA3584	Deletion	CoNIFER	chr2:228,678,570-228,789,026	110,456	0	0	*CCL20, DAW1, SPHKAP*	Rheumatoid arthritis–*CCL20* (#601960); axonemal dynein –DAW1
		ExomeDepth	chr2:228,678,628-228,860,410	181,782	0	0		
AA3584	Duplication	CoNIFER	chr7:84,624,869-84,751,247	126,378	0	0	*SEMA3D*	Axon guidance (#609907)
		ExomeDepth	chr7:84,685,033-84,727,281	42,248	0	0		
AA3584	Duplication	CoNIFER	chr11:20,691,136-21,597,001	905,865	0	0	*NELL1*	Osteogenesis (#602319)
		ExomeDepth	chr11:21,555,920-21,596,568	40,648	0	0		
AA3589	Duplication	CoNIFER	chr5:140,529,839-140,555,957	26,118	0	1	*PCDHB6, PCDHB17, PCDHB7, PCDHB8*	Neural cadherins–*PCDHB7* and *PCDHB8* (#606333 and #606334)
		ExomeDepth	chr5:140,552,417-140,560,021	7604	9	0		
AA3589	Duplication	CoNIFER	chr9:117,085,336-117,088,757	3421	0	0	*ORM1*	Immunosuppression (#138600)
		ExomeDepth	chr9:117,085,943-117,087,432	1489	0	0		
AA3589	Deletion	CoNIFER	chr15:43,891,761-43,941,039	49,278	4	0	*CKMT1B, STRC, CATSPER2*	Mitochondrial creatine kinase–*CKMT1B* (#123290); deafness–*STRC* (#606440)
		ExomeDepth	chr15:43,888,606-43,897,597	8991	0	0		
U726	Duplication	CoNIFER	chr9:117,085,336-117,088,757	3421	0	0	*ORM1*	Immunosuppression (#138600)
		ExomeDepth	chr9:117,085,414-117,087,432	2018	0	0		

Abbreviations: bp, base pairs; CNV, copy number variant; DGV, Database of Genomic Variants; Freq., frequency; OMIM, Online Mendelian Inheritance in Man.

Table S2. List of genes where a potentially pathogenic germline SNV/indel and tumor LOH were identified in our samples.

Gene	Sample	Chromosome	Genomic Position	Reference Allele	Alternative Allele	Pathogenicity Tools	ExAC Freq.
ABCA12	AA3588	2	215,854,157	G	A	5/6	1.58E-05
ACADVL	AA3588	17	7,127,641	C	T	3/6	6.32E-05
AMOT	AA3588	X	112,022,894	C	T	3/6	6.87E-04
ANAPC5	AA3588	12	121,773,343	C	T	6/6	1.58E-05
ANO7	AA3588	2	242,144,358	C	A	NS	-
AREL1	AA3588	14	75,136,366	C	T	6/6	-
ARHGEF25	AA3584	12	58,008,801	G	C	SP	-
ARMC3	AA3584	10	23,321,876	T	C	3/6	2.53E-04
ASPM	AA3584, AA3582	1	197,073,396	A	C	5/6	1.11E-04
ASPN	AA3588	9	95,221,948	A	AT	FS	7.42E-04
ASTN1	AA3585	1	176,851,995	A	T	3/6	-
ATCAY	AA3585	19	3,907,776	G	A	4/6	3.99E-05
ATP4A	AA3588	19	36,046,582	G	A	4/6	1.97E-04
ATP7B	AA3583	13	52,515,271	C	T	3/6	5.58E-05
B4GALNT3	U729	12	670,538	G	A	6/6	8.68E-05
	AA3585	12	655,811	G	T	SP	5.74E-05
BCL9	AA3585	1	147,084,974	C	G	5/6	-
BLM	U729	15	91,306,382	C	T	6/6	7.89E-06
BORA	AA3585	13	73,319,166	C	A	6/6	2.96E-04
BRCA2	AA3599	13	32,913,453	GT	G	FS	-
CELSR1	AA3583	22	46,835,164	G	A	3/6	1.58E-05
CELSR2	AA3588	1	109,811,556	G	A	3/6	7.89E-05

CELSR3	AA3585	3	48,692,507	G	A	5/6	7.89E-06
	AA3589	3	48,688,833	C	T	4/6	1.11E-04
CLCA2	AA3588	1	86,913,377	C	T	6/6	1.82E-04
CNTNAP2	AA3588	7	147,259,253	G	A	4/6	-
COBLL1	AA3588	2	165,578,715	G	A	3/6	-
COL1A1	AA3586	17	48,270,048	C	T	3/6	1.74E-04
	AA3584	17	48,264,061	G	A	5/6	7.89E-06
COL6A3	AA3585	2	238,277,797	T	A	3/6	3.95E-05
COL9A3	AA3585	20	61,461,143	G	A	4/6	6.42E-05
COLEC12	AA3588	18	348,074	T	C	4/6	7.89E-06
CSMD3	AA3588	8	113,326,269	T	C	3/6	9.79E-04
CST6	AA3588	11	65,779,570	C	T	3/6	-
DCN	AA3588	12	91,545,375	AGGGTG	A	FS	7.10E-05
DHRS2	AA3585	14	24,108,386	A	G	SP	1.58E-05
DLG5	AA3584	10	79,580,910	C	T	3/6	1.58E-05
DNASE1	AA3589	16	3,706,649	G	A	6/6	4.82E-04
DOPEY1	U729	6	83,877,666	T	C	4/6	5.53E-05
DPT	AA3582	1	168,698,123	C	T	5/6	-
DST	AA3599	6	56,497,777	A	C	4/6	-
DYNC1H1	AA3588	14	102,500,421	C	A	6/6	1.50E-04
ECT2	AA3586	3	172,480,522	C	G	3/6	3.16E-05
ELK4	AA3582	1	205,589,296	G	C	4/6	9.47E-05
EPB41L4B	AA3596	9	112,017,891	A	C	5/6	-
EPPK1	AA3600	8	144,946,135	CTGAG	C	FS	8.05E-04

ERCC2	AA3584	19	45,867,712	C	T	4/6	-
FAT2	AA3600	5	150,946,850	A	G	5/6	1.58E-05
FDFT1	AA3582	8	11,679,329	G	A	6/6	3.16E-05
FERMT2	AA3582	14	53,417,220	G	A	3/6	3.08E-04
FGF12	AA3584	3	191,861,866	G	A	5/6	-
FMOD	AA3582	1	203,317,176	C	T	4/6	3.95E-05
FOXJ3	AA3582	1	42,657,223	C	T	3/6	4.34E-04
GNRHR	AA3585	4	68,619,623	G	A	6/6	7.89E-06
GPR110	AA3596, AA3582	6	46,976,836	TCA	T	FS	4.74E-05
GPR98	AA3582	5	90,059,209	G	A	3/6	8.81E-05
HCK	AA3598	20	30,671,808	C	T	6/6	7.89E-06
HEXIM1	AA3551	17	43,226,669	G	A	3/6	1.50E-04
HEY1	AA3588	8	80,679,521	C	A	3/6	-
HOOK3	AA3584	8	42,841,856	T	G	3/6	7.10E-05
HOXD4	AA3589	2	177,016,498	TC	T	FS	-
HSPG2	AA3551	1	22,202,391	C	T	3/6	3.16E-05
HUNK	AA3582	21	33,370,865	C	T	5/6	6.32E-05
IFT172	AA3585	2	27,672,571	C	T	6/6	1.34E-04
IGF2R	AA3589	6	160,412,298	G	A	6/6	7.89E-06
IL7R	AA3588	5	35,867,562	A	C	4/6	8.68E-05
ITGA4	AA3588	2	182,396,457	C	A	4/6	7.83E-04
ITIH5	AA3551	10	7,682,770	C	G	4/6	6.47E-04
KCNH5	AA3588	14	63,174,240	C	A	NS	1.06E-04
KNTC1	AA3589	12	123,068,956	T	C	6/6	1.28E-04

KRT23	AA3583	17	39,087,671	G	T	3/6	-
LATS2	AA3585	13	21,619,829	C	T	5/6	7.89E-06
LILRB2	U729	19	54,779,854	G	A	3/6	1.97E-04
LMO7	U726	13	76,335,093	C	G	5/6	-
LONP1	AA3583	19	5,705,801	T	C	3/6	4.74E-05
LTBP3	AA3598	11	65,315,172	C	A	4/6	-
MAML1	AA3582	5	179,193,441	C	A	4/6	1.74E-04
MAP3K4	AA3585	6	161,470,614	G	T	4/6	5.21E-04
MAST2	AA3551	1	46,496,378	G	T	3/6	2.55E-04
MCF2L	AA3588	13	113,744,418	C	T	3/6	-
MELK	AA3589	9	36,651,798	G	A	5/6	5.53E-05
MLLT4	AA3585	6	168,316,012	T	A	5/6	-
MYO1E	AA3584	15	59,510,112	C	T	6/6	2.37E-05
NID1	AA3585	1	236,145,007	C	T	4/6	7.89E-06
NISCH	AA3588	3	52,505,834	A	T	3/6	3.63E-04
NME7	AA3589	1	169,292,503	G	A	5/6	7.89E-06
NR1D1	AA3588	17	38,252,065	G	A	5/6	2.37E-05
NR3C2	U726	4	149,356,823	T	C	4/6	7.89E-06
NRCAM	AA3598	7	107,834,771	G	A	3/6	7.89E-06
NUP160	AA3598	11	47,840,937	G	C	5/6	1.50E-04
PARP2	AA3599	14	20,823,075	G	C	3/6	2.39E-05
PCDHB1	AA3584	5	140,433,067	A	T	5/6	7.89E-06
PCDHGA8	AA3596	5	140,773,936	C	T	4/6	1.58E-05
PDE1B	AA3584	12	54,968,980	T	G	6/6	-

PHGDH	AA3582	1	120,263,814	C	T	5/6	7.89E-06
PHKA2	AA3582	X	18,924,895	C	T	5/6	2.37E-05
PHRF1	AA3586	11	607,593	CGACT	C	FS	-
PI4K2A	AA3584	10	99,426,866	A	C	5/6	-
PIK3R3	AA3585	1	46,597,560	T	C	5/6	1.34E-04
PLCD3	AA3597	17	43,195,480	C	T	6/6	3.18E-05
PLEC	AA3584	8	144,998,705	C	T	3/6	1.85E-05
PLXND1	AA3598	3	129,308,229	C	G	5/6	1.11E-04
POSTN	AA3584	13	38,153,449	C	T	6/6	2.37E-05
PPFIA2	AA3582	12	81,734,962	C	T	4/6	8.00E-06
PREX2	U729	8	69,009,359	G	A	5/6	1.58E-05
PRRC2A	AA3584	6	31,592,082	C	A	3/6	9.55E-04
PSMD9	AA3583	12	122,337,659	A	T	3/6	2.37E-04
PSRC1	AA3551	1	109,823,551	G	A	4/6	6.31E-05
PTK6	AA3585	20	62,168,601	TC	T	FS	1.37E-04
PTPN14	AA3585	1	214,557,352	C	T	6/6	8.68E-05
PYGO1	AA3583	15	55,838,573	G	A	5/6	3.16E-05
RASSF6	AA3585	4	74,447,572	G	A	6/6	4.34E-04
RECQL	AA3589	12	21,643,302	C	A	6/6	-
	AA3589	12	21,643,306	G	T	4/6	-
RERGL	AA3589	12	18,234,381	A	G	6/6	4.58E-04
REV3L	AA3597	6	111,726,679	T	A	5/6	-
RIF1	AA3585	2	152,320,296	G	A	4/6	3.95E-05
RREB1	AA3586	6	7,231,360	G	C	5/6	7.12E-05

RRP12	AA3584	10	99,129,270	C	T	6/6	5.53E-05
SALL3	AA3582	18	76,757,135	C	T	5/6	-
SEC23B	AA3588	20	18,505,241	G	C	4/6	7.89E-06
SERPINB10	AA3585	18	61,587,044	C	T	3/6	6.79E-04
SHMT1	AA3585	17	18,232,669	C	T	6/6	4.74E-05
SIK3	AA3596	11	116,734,472	C	T	5/6	2.37E-05
SLC33A1	AA3582	3	155,571,417	G	A	5/6	2.68E-04
SLIT3	AA3582	5	168,199,939	G	C	6/6	-
SMARCA4	AA3597	19	11,096,021	C	T	5/6	7.89E-06
SORL1	AA3598	11	121,456,987	C	T	5/6	-
SPDL1	AA3584	5	169,021,633	A	G	5/6	4.58E-04
SPEG	AA3584	2	220,355,229	T	G	3/6	3.19E-04
SPTBN1	AA3585	2	54,895,645	G	T	3/6	-
STK11IP	AA3588	2	220,471,854	C	T	5/6	4.08E-04
TAF6	AA3582	7	99,708,920	T	A	3/6	-
TENC1	AA3586	12	53,453,362	C	T	3/6	5.53E-05
TENM2	AA3582	5	167,674,669	G	A	6/6	1.59E-05
TIAM2	AA3588	6	155,485,700	T	C	6/6	7.89E-06
TMBIM1	AA3600	2	219,140,257	A	G	6/6	1.74E-04
TMC6	AA3584	17	76,121,897	G	A	4/6	5.16E-05
TNRC6A	AA3600	16	24,800,913	A	G	4/6	8.68E-05
TRAP1	AA3598	16	3,708,209	G	A	3/6	7.89E-06
TRPM8	AA3551	2	234,854,632	C	T	6/6	3.95E-05
	AA3588	2	234,869,523	C	T	6/6	6.39E-04

VWA5A	AA3589	11	124,005,718	G	A	3/6	5.53E-05
YLPM1	AA3582	14	75,265,235	C	T	4/6	1.43E-04
ZFAND4	AA3584	10	46,122,195	A	T	5/6	8.45E-04
ZNF521	AA3582	18	22,804,982	C	T	4/6	3.95E-05

Abbreviations: ExAC, Exome Aggregation Consortium; Freq., frequency; FS, frameshift; NS, nonsense variant; SP, splicing-affecting variant.

Table S3. List of potentially pathogenic rare germline variants located in the final candidate genes in a cohort of 1006 familial early onset CRC patients from the CanVar database. Only frameshift or missense variants with CADD > 15 were selected. Significant variant enrichment in cases compared to ExAC control database (*p*-value < 0.05) have been marked in bold.

Gene	Genetic Variant	CADD	CanVar Freq.	ExAC Freq.	*p*-Value
ADCY8	p.Asp209Glu	23.4	0.00105	0.00002	**0.002**
	p.Gln320Glu	25.5	0.00060	-	**0.016**
	p.Ile675Val	25.5	0.00103	0.00002	**0.002**
	p.Val722Ile	18.83	0.00100	0.00008	**0.013**
	p.Ser812Leu	23.7	0.00050	0.00017	0.304
	p.Ala878Thr	16.76	0.00051	0.00007	0.138
	p.Asp893PhefsTer46	FS	0.00051	-	**0.016**
	p.Arg924Cys	33	0.00100	0.00002	**0.002**
BLM	p.Leu9Pro	26.2	0.00050	-	**0.016**
	p.Cys361Ter	FS	0.00050	-	**0.016**
	p.Glu880Gln	22.5	0.00050	0.00003	0.064
	p.Gly1359Glu	24.3	0.00061	-	**0.016**
BRCA2	p.Ile505Thr	18.18	0.00204	0.00071	0.060
	p.Leu1227GlnfsTer5	FS	0.00051	-	**0.016**
	p.Ser1230LeufsTer9	FS	0.00050	-	**0.016**
	p.Gly1529Arg	27.0	0.00050	0.00040	0.553
	p.Lys1690Asn	23.7	0.00050	0.00013	0.231
	p.Tyr1710Ter	FS	0.00050	-	**0.016**
	p.Leu2092ProfsTer7	FS	0.00050	0.00002	**0.048**
	p.Val2179AspfsTer10	FS	0.00050	0.00001	**0.032**
	p.Glu2856Ala	25.9	0.00550	0.00078	**<0.001**
	p.Thr3013Ile	22.1	0.00050	0.00022	0.358
	p.Tyr3035Cys	27.4	0.00053	0.00003	0.079
	p.Tyr3035Ser	26.9	0.00053	0.00006	0.123
	p.Leu3274Trp	28.7	0.00051	-	**0.016**
ERCC2	p.Arg143Gly	25.2	0.00053	0.00001	**0.032**
	p.Phe610LeufsTer99	FS	0.00052	0.00001	**0.032**
	p.Ala717Gly	24.8	0.00054	0.00033	0.490
HSPG2	p.Glu113Lys	24.2	0.00205	0.00018	**0.001**
	p.Gln372Arg	22.9	0.00109	0.00005	**0.007**
	p.Val376Ile	23.5	0.00052	0.00007	0.138
	p.Arg420Gln	32	0.00054	0.00025	0.409
	p.Asp746Val	20.7	0.00053	0.00001	**0.032**
	p.His779Tyr	23.3	0.00374	0.00094	**0.001**
	p.Asp802Tyr	29.0	0.00052	0.00001	**0.032**
	p.Ala803Thr	25.1	0.00052	0.00006	0.123
	p.Arg940Cys	32	0.00058	-	**0.016**
	p.Thr1182Met	25.1	0.00055	0.00004	0.094
	p.Glu1526Lys	29.2	0.00073	0.00002	0.064
	p.Val1736Ile	18.67	0.00053	0.00037	0.523
	p.Arg1758Gln	25.6	0.00052	0.00005	0.109

	p.Arg1779Trp	29.3	0.00054	0.00014	0.256
	p.Val1867Met	15.75	0.00058	-	**0.016**
	p.Ala1883Val	24.5	0.00114	0.00058	0.329
	p.Ala2164Val	22.5	0.00057	0.00002	**0.048**
	p.Gly2270Arg	29.7	0.00208	0.00026	**0.003**
	p.Arg2377His	25	0.00054	0.00009	0.179
	p.Ser2412Asn	16.42	0.00114	0.00072	0.658
	p.Leu2459His	25.5	0.00054	-	**0.016**
	p.Val2738Met	24.2	0.00050	0.00004	0.094
	p.Val3079Met	21.9	0.00160	0.00030	**0.027**
	p.Arg3159Gln	16.31	0.00057	0.00023	0.379
	p.Gln3188His	21.2	0.00267	0.00054	**0.006**
	p.Arg3334His	24.5	0.00059	0.00006	0.123
	p.Ala3396Val	15.38	0.00054	0.00024	0.389
	p.Leu3451Phe	28.8	0.00057	0.00040	0.553
	p.Pro3487His	26.7	0.00066	0.00020	0.337
	p.Gly3934Arg	26.2	0.00063	0.00002	**0.048**
	p.Arg4086Trp	33	0.00052	0.00061	1.000
PARP2	p.Val163Met	22.3	0.00050	0.00010	0.206
RECQL	p.Ile497AsnfsTer12	FS	0.00051	0.00008	0.152
	c.1667_1667+3delAGTA	SP	0.00051	0.00035	0.515
REV3L	p.Gln578Lys	22.2	0.00050	0.00001	**0.032**
	p.Trp1129Cys	32	0.00051	0.00002	**0.048**
	p.Asp1202Asn	22.7	0.00149	0.00034	**0.035**
	p.Cys1442Tyr	16.68	0.00050	-	**0.016**
RIF1	p.Arg116Cys	22.1	0.00298	0.00021	**<0.001**
	p.Ile224Val	15.61	0.00104	0.00043	0.220
	p.Pro403Leu	22.2	0.00050	0.00039	0.546
	p.Lys1303Asn	17.19	0.00249	0.00053	**0.005**
	p.Glu1598Ala	15.30	0.00050	0.00001	**0.032**
	p.Asp2378Glu	16.52	0.00050	-	**0.016**
SEC23B	p.Arg546Trp	26.1	0.00165	0.00005	**<0.001**
SMARCA4	p.Arg359Gln	16.81	0.00183	0.00020	**0.001**
STK11IP	p.Arg550Cys	24.3	0.00056	0.00001	**0.032**
	p.Arg1061Cys	28.1	0.00055	0.00002	**0.048**

Abbreviations: CADD, combined annotation dependent depletion; ExAC, Exome Aggregation Consortium; Freq., frequency.

© 2019 by the authors. Licensee MDPI, Basel, Switzerland. This article is an open access article distributed under the terms and conditions of the Creative Commons Attribution (CC BY) license (http://creativecommons.org/licenses/by/4.0/).
